# Impaired autophagy and lysosome function in human fibroblast aging

**DOI:** 10.3389/fragi.2026.1971981

**Published:** 2026-10-14

**Authors:** Juan José Unzueta-Mendoza, Ian García-Aguirre, Mina Königsberg, Isabel Arrieta-Cruz, Solangy Lizcano-Meneses, Jonathan J. Magaña, Adriana Alarcón-Aguilar, Bulmaro Cisneros

**Affiliations:** 1 Posgrado en Biología Experimental, División de Ciencias Biológicas y de la Salud, Universidad Autónoma Metropolitana Unidad-Iztapalapa, Ciudad de México, Mexico; 2 Laboratorio de Bioenergética y Envejecimiento Celular, División de Ciencias Biológicas y de La Salud, Universidad Autónoma Metropolitana Unidad-Iztapalapa, Ciudad de México, Mexico; 3 Departamento de Genética y Biología Molecular, Centro de Investigación y de Estudios Avanzados, Ciudad de México, Mexico; 4 Departamento de Bioingeniería, Escuela de Ingeniería y Ciencias, Tecnológico de Monterrey, Ciudad de México, Mexico; 5 Departamento de Investigación Básica, División de Investigación, Instituto Nacional de Geriatría, Secretaría de Salud, Ciudad de México, Mexico; 6 Departamento de Biología Celular, Centro de Investigación y de Estudios Avanzados, Ciudad de México, Mexico; 7 Laboratorio de Medicina Genómica, Departamento de Genética, Instituto Nacional de Rehabilitación-Luis Guillermo Ibarra (INR-LGII), Ciudad de México, Mexico

**Keywords:** aging, autophagosomes, autophagy flux, fibroblast aging, lysosomes

## Abstract

**Background:**

Human aging is characterized by a progressive, time-dependent deterioration in physiological and cognitive functioning. Aging is driven by an array of interconnected cellular hallmarks, including genomic instability, mitochondrial dysfunction, telomere attrition, epigenetic alteration and dysfunctional autophagy. Autophagy is a cytoprotective (cellular degradation and recycling) pathway that maintains homeostasis during starvation or stress, and whose activity is associated with extended lifespan. This multi-step process comprises autophagosome formation and maturation, autophagosome–lysosome fusion, and cargo degradation. Thus, analysis of autophagy flux is necessary to precisely design therapeutic interventions aimed at restoring autophagy in aging.

**Methods:**

The autophagy flux was comprehensively analyzed in human skin fibroblasts from donors of different ages (20–96 years old), a cell system that retains age-associated cellular and molecular signatures. The subcellular localization and activity of the master transcription factor of autophagy and lysosome biogenesis, TFEB, was analyzed by confocal microscopy assays and transcriptional expression of TFEB target genes (RT-qPCR) respectively. The autophagy flux was evaluated by monitoring the autophagy markers LC3 and p62 by Western blotting and confocal microscopy analyses, using inhibitor drugs to block autophagy or lysosomal activity. Autophagosome maturation and autophagosome-lysosome fusion were analyzed by colocalizing LC3 with Sintaxin-17 and LC3 with the lysosomal protein LAMP1 respectively, using inhibitor drugs and confocal microscopy assays. The analysis of lysosomal morphology and function was evaluated by lysobrite dye staining and live-cell confocal microscopy analysis.

**Results:**

The initial stages of autophagy, including activation of TFEB, and autophagosome formation, remained apparently functional in fibroblast aging. In contrast, the rate of both autophagosome maturation and autophagosome-lysosome fusion, as well as the lysosomal function appears to be altered in aged fibroblasts. Specifically, enlarged dysfunctional lysosomes were identified as a hallmark of the fibroblasts of the 96-year-old donor. This lysosomal dysfunction may lead to a reduced ability to degrade canonical autophagy markers, LC3-II and p62, and the accumulation of lipofuscin.

**Conclusion:**

Overall, the results provide evidence that autophagy flux is impaired at the late stages in aged fibroblasts, specifically in autophagosome maturation and autophagosome-lysosome fusion steps. Furthermore, it was demonstrated that this failure correlates with the presence of enlarged dysfunctional lysosomes. This study underscores the significance of enhancing lysosomal activity to counteract autophagy decline in aging.

## 1 Introduction

Aging is a complex, progressive, and irreversible process that arises from a series of alterations in the functioning of cells and tissues. At the systemic level, these alterations result in the decline of the whole physiology of the individual and the progressive loss of cognitive function (López-Otin et al., 2023). This state of vulnerability, which encompasses the immune system, predisposes individuals to a higher risk of developing chronic degenerative diseases, including cancer, cardiovascular disorders, diabetes, and neurodegenerative diseases, and ultimately results in mortality (Guo et al., 2022). Over the past two decades, a substantial body of research on the biology of aging has identified key cellular and molecular drivers of aging, termed the “hallmarks of aging”, which, in turn, has enabled the design of therapeutic interventions aimed at halting or decelerating age-related pathologies and improving healthspan. These distinctive hallmarks include, among others, genomic instability, telomere attrition, epigenetic alterations, loss of proteostasis, mitochondrial dysfunction, cellular senescence, and, more recently, disabled macroautophagy (Guo et al., 2022; López-Otin et al., 2023; Tartiere et al., 2024).

Macroautophagy (hereafter referred to as autophagy) is a fundamental catabolic and renewal cellular process that serves as a quality control mechanism. The initiation of autophagy is regulated at the level of transcription by the master transcription factor EB (TFEB), a key regulator of autophagy and lysosomal genes (Feng et al., 2014; Gómez-Virgilio et al., 2022). Subsequently, the process involves the enclosure of damaged organelles, misfolded proteins, and intracellular pathogens within double-membrane, specialized vesicles termed autophagosomes (Feng et al., 2014; Gómez-Virgilio et al., 2022). Autophagosomes are subsequently transported to and fuse with lysosomes, where the luminal content is degraded by lysosomal hydrolases, resulting in the recycling of building blocks to produce new cellular structures (Feng et al., 2014; Gómez-Virgilio et al., 2022). Autophagy dysfunction is implicated in aging as well as in a variety of diseases, including cancer and neurodegenerative diseases (e.g., Alzheimer’s and Parkinson’s diseases) (Gómez-Virgilio et al., 2022; Nakamura and Yoshimori, 2018). The accumulation of damaged organelles and misfolded protein aggregates is linked to severe impairment of cellular homeostasis and overall health (Gómez-Virgilio et al., 2022; Nakamura and Yoshimori, 2018; Palmer et al., 2025; Parzych and Klionsky, 2014). A significant body of research in both cell and animal models has demonstrated a correlation between impaired autophagy and aging. For instance, the transcriptional expression of several autophagy-related genes (ATG) was found to decrease in the brain of *Drosophila* (*Atg2, Atg8A, and Atg18*), and in human brain samples of aged individuals (ATG7, ATG52, and ATG16L2) (Lipinski et al., 2010; Simonsen et al., 2008). Furthermore, decreased protein content of LC3 (Microtubule-associated protein light chain 3) and ATG7 was observed in the muscle of aged mice (Carnio et al., 2014). In other experiments, the inhibition of autophagy has been shown to accelerate aging and reduce lifespan in worms, flies, and mice (Nakamura et al., 2019). Opposing this, activation of autophagy by overexpressing autophagy genes (Atg5 and SQST-1) has been demonstrated to improve healthspan in *Drosophila and C. elegans* (Fernández et al., 2018; Kumsta et al., 2019; Pyo et al., 2013). Autophagy flux is subdivided into several phases: formation of a phagophore upon starvation signaling that inhibits mTOR and activates ULK1/2 complex (Unc-51-like autophagy activating kinase ½); nucleation and elongation of the phagophore by two ubiquitin-like conjugation systems (ATG12/ATG5/ATG16L and LC3; formation of autophagosomes by the expansion and closure of the phagophore; maturation of autophagosomes and its SNARE (Soluble N-ethylmaleimide-sensitive factor activating protein receptors)-mediated fusion of autophagosomes with lysosomes to form autolysosomes; and degradation of the luminal content by lysosomal hydrolases (Parzych and Klionsky, 2014). Thus, a comprehensive examination of autophagy flux will enable the development of a more precise design to reactivate a specific phase that is altered in aging.

The aim of this study was to analyze autophagy flux in an aging cell system based on primary dermal fibroblasts from different healthy individuals with an age range that reflects aging (20–96 years). This aging cell model is a widely adopted paradigm for studying cellular and molecular changes associated with aging (Brun et al., 2016; Cristofalo et al., 1998; Kim et al., 2018; Rorteau et al., 2022; Tigges et al., 2014; Tsitsipatis et al., 2022; Zorin et al., 2019). This paradigm is based on the notion that the skin is directly exposed to external stressors, including UV radiation, environmental pollutants, and genotoxic agents, which cause damage to accumulate over a lifetime. Thus, primary dermal fibroblasts form aged individuals exhibit the *in vivo* aging characteristics of their donors, providing a reliable representation of the donor’s chronological age within a controlled *in vitro* environment (Heckmann and Green, 2019; Heckmann et al., 2019; Itakura et al., 2012; Itakura and Mizushima, 2013; Keshri et al., 2024; Kim et al., 2018; Kumsta et al., 2019). The collective evidence from this study indicates that autophagy flux is impaired in aged fibroblasts at the late steps, namely, autophagosome maturation and autophagosome-lysosome fusion. We propose that this impairment is partly due to the presence of enlarged dysfunctional lysosomes.

## 2 Materials and methods

### 2.1 Cell culture and treatments

The following human dermal fibroblast cultures were utilized: Skin primary fibroblasts from apparently healthy men donors aged 20 years (GM03440), 38 years (AG08469), 74 years (AG06290), and 96 years (AG04059) were obtained from the Coriell Institute for Medical Research (Camden, NJ). Fibroblast cultures were grown in Minimal Essential Medium Eagle (MEM; Invitrogen, Carlsbad, CA), with the following supplements: 10% fetal bovine serum (FBS; Invitrogen, United States), 1 mM sodium pyruvate, antibiotics, and antimycotics (Sigma, Saint Louis, MO). Cultures were maintained at 37 °C in a humidified 5% CO_2_ atmosphere incubator. In conditions of starvation, fibroblast cultures were grown in Krebs-Ringer-Bicarbonate (KRB) buffer for 1 or 4 h (Sigma, Saint Louis, MO). When indicated, fibroblast cultures were treated with 50 µM chloroquine (CQ) (Sigma, Saint Louis, MO; C6628) for 24 h or with 50 µM Bafilomycin A1 (BafA1; Sigma, Saint Louis, MO; SML1661) for 12 h to halt autophagy or incubated with 10 μg/mL E64D (SC201280A, Santa Cruz Biotechnology, CA) and 10 μg/mL pepstatin A (P5318, Sigma, Saint Louis, MO) for 4 h to inhibit lysosomal activity. Cultures were then subjected to immunofluorescence (IF) and Western blot (WB) analyses. All experiments used donor-matched cell cultures, restricted to passages 4–10 to maintain biological comparability across cohorts.

### 2.2 Antibodies

The following primary antibodies were used: rabbit polyclonal and monoclonal antibodies against LC3 (MBL International, Woburn, MA [PM036]); LAMP1 (Sigma, Saint Louis, MO [L1418-200]); STX17 (Invitrogen, Carlsbad, CA [PA5-40127]); TFEB (Cell Signaling Technology, MA [4240]); SQSTM1/p62 (Abcam, Cambridge, UK [Ab109012]). Mouse monoclonal antibodies against β-actin (a gift from Dr. Manuel Hernández, CINVESTAV, Mexico).

### 2.3 Indirect immunofluorescence and confocal microscope analysis

Cells were seeded on coverslips and fixed with 4%paraformaldehyde (PFA) in phosphate-buffered saline (PBS) for 10 min paraformaldehyde (PFA) in phosphate-buffered saline (PBS) for 10 min. Thereafter, they were permeabilized and blocked for 10 min at room temperature with immunofluorescence (IF) buffer containing 0.1% Triton X-100, 0.02% SDS, and 10 mg/mL BSA. Then, the cells were incubated at 4 °C overnight with the corresponding primary antibodies. The following day, the cells were washed twice with PBS for 5 min, and then, incubated for 1 h at room temperature with the appropriate fluorochrome-conjugated secondary antibodies (Jackson Immuno Research Laboratories). For double staining, cells were subjected to an overnight incubation at 4 °C with the appropriate primary antibody. The following day, the cells were washed with phosphate-buffered saline (PBS) and incubated for one hour at room temperature with secondary Alexa Fluor 594 chicken anti-goat IgG (1:25, Invitrogen). The cells were then processed for double labeling by incubating overnight at 4 °C with the appropriate second primary antibody. The following day, the cells were incubated with secondary FITC-conjugated goat anti-rabbit IgG (1:25; Zymed Laboratories, Inc., San Francisco, CA, United States) for 1 h at room temperature. Subsequently, the cells were incubated for five minutes at room temperature with DAPI (0.2 μg/mL diamino-2-phenylindole; Sigma-Aldrich) in PBS for nuclei labeling. After washing twice with PBS for 5 min, the coverslips were mounted on microscope slides using VectaShield (Vector Laboratories, Inc., Burlingame, CA) and further examined on a confocal laser scanning microscope (CLSM; Eclipse Ti-E inverted confocal laser scanning microscope, NiKon, Japan). The analysis of the digitized images was performed using ImageJ2 software, version: 2.14.0/1.54f (http://imageJ.nih.gov.ij).

### 2.4 Western blotting

The fibroblasts were resuspended in RIPA (Radioimmunoprecipitation assay) 1X buffer containing 1X Protease/Phosphatase inhibitor (Cell Signaling Technology, MA [5872S]) and 1% SDS. The lysates were obtained by sonication for 5 min and subsequently centrifugation at 14,000 rpm at 4 °C for 10 min. Lysate samples were subjected to vertical electrophoresis on 10% or 15% (LC3 analysis) SDS-polyacrylamide gels and transferred onto nitrocellulose membranes (Bio-Rad Laboratories, Hercules CA) using a semi-dry transblot apparatus (Bio-Rad Laboratories, Hercules CA) for 1 h or 15 min (for LC3 detection) at 20 V. The membranes were blocked in TBST (100 mM Tris-HCl pH 8.0, 150 mM NaCl, 0.5% (v/v) Tween-20) with 5% low-fat dried milk for 1 h at room temperature and further incubated at 4 °C overnight with the appropriate primary antibodies. Subsequently, the protein signal was developed using horseradish peroxidase-conjugate secondary antibodies (Sigma-Aldrich, MO, United States) and the enhanced chemiluminescence Western blotting detection system (Western Lightning, Plus ECL, Netherlands [NEL 104001EA]) according to the manufacturer´s instructions. The acquisition of the blot images was performed using the C-Digit® Blot Scanner (LI-COR Biosciences, Lincoln, Nebraska). The digitized images were then subjected to analysis using Image Studio software, version: 5.2.5 (LI-COR Biosciences, Lincoln, Nebraska).

### 2.5 Total RNA isolation and reverse transcription-quantitative polymerase chain reaction (RT-qPCR) assays

Total RNA was obtained from human dermal fibroblasts using TRIzol Reagent (Invitrogen; Thermo Fisher Scientific, Inc., MA, United States) in accordance with the manufacturer’s instructions. The RNA was quantified using a NanoDrop 2000 Spectrometer (Invitrogen; Thermo Fisher Scientific, Inc., MA, United States). One-step RT–PCR was performed using KAPA SYBR Fast One-Step Universal (cat. no. KK4651; Roche, Inc.) on a StepOnePlus Real-Time PCR System (Applied Biosystems; Thermo Fisher Scientific, Inc.). (Invitrogen; Thermo Fisher Scientific, Inc., Waltham, MA, United States). The relative mRNA expression of the indicated genes was analyzed using the 2−ΔΔCq method and the following primers. *Lysosomal-associated membrane protein 1 (*LAMP1): forward, 5′-AAC​GGG​ACC​GCG​TGC​GAT​ATG-3′ and reverse, 5′-GGT​TGA​GCA​CCA​CTG​TGG​CAT​CT-3’. Sequestosome-1 (SQSTM1): forward, 5′-CGG​AAG​GTG​AAA​CAC​GGC​ACT-3′ and reverse, 5′-CTC​CGA​TGG​ACC​GAA​AGC​TGA​T-3’. Microtubule-associated protein 1 light chain 3 beta (MAP1LC3B): forward, 5′-GTC​CGA​CTT​ATT​CGA​GAG​CAG​C-3′ and reverse, 5′-GGC​CTG​ATT​AGC​ATT​GAG​CTG​T-3’. Hypoxanthine phosphoribosyltransferase 1 gene (HPRT): forward: 5′- GAT​GAA​CCA​GGT​TAT​GAC​CT-3′ and reverse: 5′-ATG​GCC​TCC​CAT​CTC​C-3’. Briefly, the PCR conditions were as follows: reverse transcription at 42 °C for 5 min and initial denaturation step at 95 °C for 3 min, followed by denaturation step at 95 °C for 3 s, annealing and extension steps at 60 °C for 20 s for 30 cycles. All experiments were performed using three independent biological triplicates and each biological sample was run in technical triplicates to minimize pipetting and instrumental variance. The HPRT gene served as the internal reference for normalization.

### 2.6 Quantification of autophagic membranes by Cyto-ID dye

To assess autophagic vacuoles (autophagosomes, and autolysosomes) in live cells, the fibroblasts were grown in p70 petri dishes (Sarstedt, Nümbrecht, Germany) until 80% confluency was reached. Thereafter, the cells were trypsinized and transferred to a 1.5-mL Eppendorf tube, to be stained with 1X of the CYTO-ID autophagy detection reagent (Enzo Life Sciences Farmingdale, NY) following the manufacturer´s instructions. Briefly, the cells were incubated for 1 h at 37 °C in the dark, then washed twice with PBS to remove the free dye and analyzed by flow cytometry at 488 nm (FL1 channel) using the BD Fortessa System. The data was plotted using Kaluza software.

### 2.7 Lysobrite red lysosome staining

The fibroblasts were seeded in 35-mm dishes with glass bottoms in MEM medium at 70% confluency and cultured for 24 h. The cells were then incubated for 30 min at 37 °C in darkness with 1x LysoBrite^TM^ Red reagent (25157, Cayman Chemical, United States) and Hoechst (1 μg/mL) for lysosome and nuclei staining respectively. Following this, the medium was removed, and the fibroblasts were washed twice with PBS. Next, the fibroblasts were observed in an *in vivo* chamber at 37 °C with 5% CO_2_. The images were captured using a laser confocal scanning microscope equipped with a ×60 objective lens (Eclipse Ti; Nikon, Japan) and processed using Fiji/ImageJ particle counter.

### 2.8 Lysosome intracellular activity

The lysosomal activity of fibroblasts was measured using a lysosomal intracellular activity kit (ab234622, Abcam, Cambridge, UK), according to the manufacturer’s instructions. Briefly, fibroblasts were grown in p70 mm Petri dishes for 12 h in MEM medium supplemented with 10% FBS at 37 °C with 5% CO_2_ The cells were then incubated in the dark with medium containing 1x self-quenched substrate for 1 h at 37 °C with 5% CO_2_. When indicated, cells were pretreated with 1x BafA1 (negative control) for 1 h. Cells were harvested and washed twice in 1 mL ice-cold 1× assay buffer and then resuspended in 300 µL PBS for analysis by flow cytometry (488-nm excitation laser) using the BD Fortessa System. The data was plotted using Kaluza software.

### 2.9 Lipofuscin assay

The fibroblasts were grown on glass coverslips and cultured for 48 h in MEM with 10% SFB medium at 37 °C with 5% CO_2_ (70%–80% confluency). Autofluorescence of lipofuscin (aggregates of oxidized proteins and lipids) exhibits broad-spectrum autofluorescence, with peak emission wavelengths of 550 and 650 nm (nm). The cells were fixed with 4% paraformaldehyde (PFA) for 10 min and mounted on glass slides using Vectashield (Vector Laboratories, Inc., Burlingame, CA) prior to being observed using an Axio Imager M2 microscope with Axiocam 506 mono camera (Carl Zeiss Microscopy GmbH, Jena, Germany) or Leica SP8 confocal microscope. A DsRed filter set was applied, with an exposure time of 500 milliseconds maintained throughout the observation. The foci of lipofuscin accumulation were subsequently measured using Fiji/ImageJ.

### 2.10 Statistical analysis

Statistical analyses were performed using GraphPad Prism 9 software (San Diego, United States) by Two-Way Analysis of Variance (Two-Way ANOVA) followed Tukey’s *post hoc* test. Data represent the mean ± standard error of the mean (SEM) from three independent experiments, and *p* values <0.05 are indicative of statistical significance.

## 3 Results

### 3.1 Analysis of the function of TFEB during human fibroblast aging

The phosphorylated, inactive form of TFEB, a key regulator of autophagy and lysosomal biogenesis (Abokyi et al., 2023; Di Malta et al., 2019), is predominantly located in the cytoplasm under physiological conditions (nutrient-rich condition). However, under conditions of stress (starvation), TFEB is activated by calcineurin-mediated dephosphorylation, resulting in its subsequent translocation to the nucleus and the upregulation of its target genes (Figure 1A) (Keshri et al., 2024; Li et al., 2019). Therefore, the protein levels, subcellular distribution and activity of TFEB were examined in cultures of fibroblast from different donor ages. TFEB was predominantly distributed in the cytoplasm, exhibiting a faint fluorescent signal within the nucleus in all fibroblast cultures, except for the 96-year fibroblasts, where the protein was predominantly localized in the nucleus.

**FIGURE 1:**
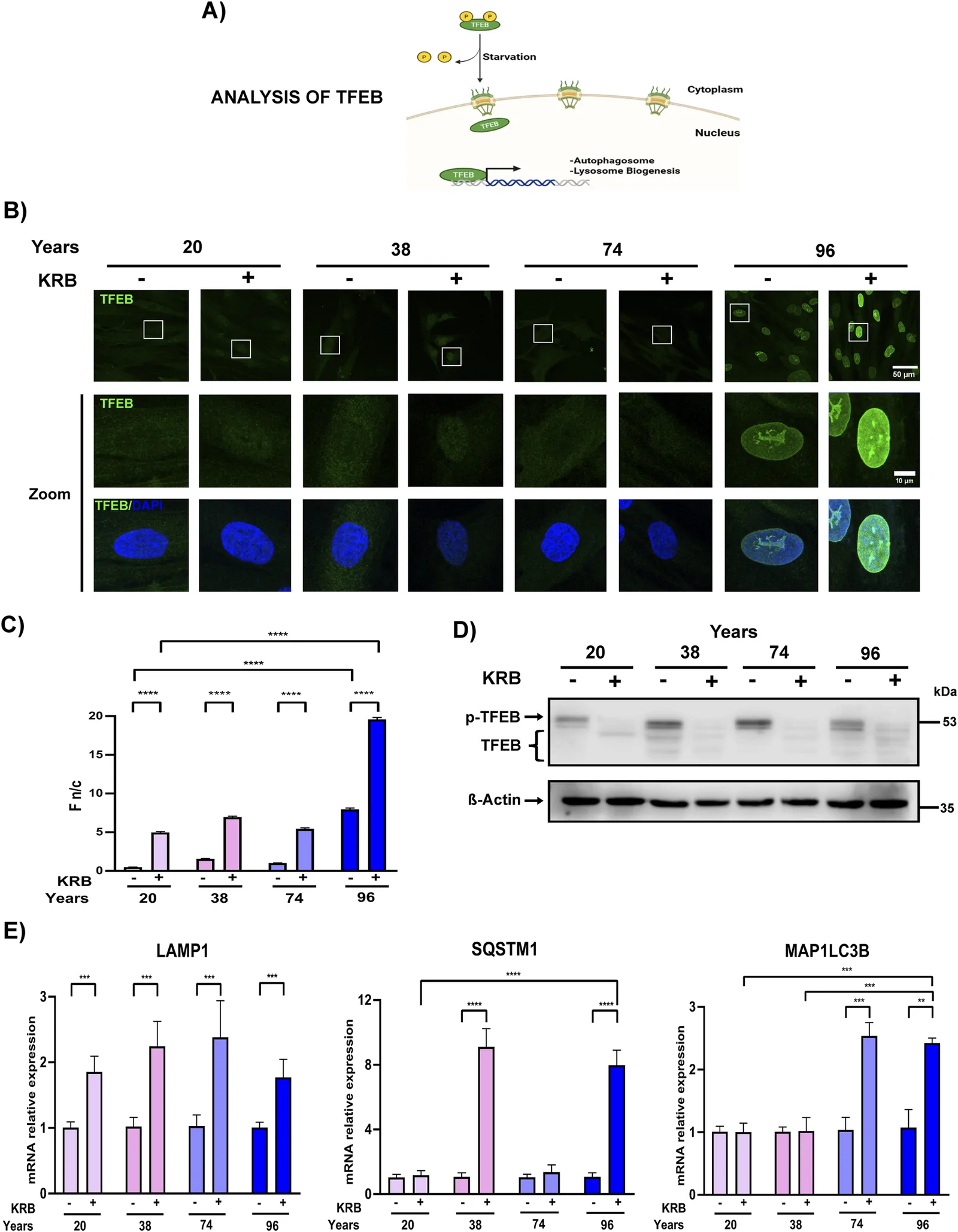
Subcellular distribution, phosphorylation state and transcriptional activity of TFEB during aging of fibroblasts. **(A)** The scheme illustrates the nuclear trafficking of TFEB under starvation conditions, which is mediated by its dephosphorylation, and its further transcriptional activity on autophagy and lysosome biogenesis genes. **(B)** Fibroblasts from donors of varying ages were cultured in MEM or KRB medium (starvation) and seeded on cover slips for 24 h, prior to be subjected to immunofluorescence analysis using primary antibodies against TFEB. The cells were counterstained with DAPI to visualize the nucleus. Typical images from three independent experiments are shown. **(C)** The cytoplasmic/nuclear fluorescence ratio (F n/c) was determined from three independent experiments using Two-Way ANOVA followed Tukey’s *post hoc* test (n = 25 cells per each independent experiment). The results are presented as the mean ± standard error of the mean (SEM), with asterisks denoting significant differences (*p ≤ 0.05; **p ≤ 0.01; ***p ≤ 0.001; ****p ≤ 0.0001). **(D)** Immunoblotting analysis of TFEB in fibroblast cultures from different donor ages using β-actin as a loading control. The migration of phosphorylated (p-TFEB) and unphosphorylated (TFEB) TFEB immunoreactive bands are indicated on the left. Typical immunoblots from three independent experiments are shown. **(E)** The relative expression level of different TFEB target genes (LAMP1, SQSTM1 and MAP1LC3B) was analyzed by quantitative polymerase chain reaction (qPCR) in total RNA samples derived from fibroblast cultures of donors varying in age, using HPRT as the internal reference gene. The results are presented as the mean ± SEM. Statistical significance between groups was determined from three independent experiments using Two-Way ANOVA followed Tukey’s *post hoc* test, with asterisks denoting significant differences (**p ≤ 0.01; ***p ≤ 0.001; ****p ≤ 0.0001).

The accumulation of TFEB increased markedly in the nucleus of all the different age-donor fibroblasts when grown in starvation conditions, with fibroblasts from a 96-year-old donor exhibiting the highest nuclear fluorescence (Figures 1B,C). On the other hand, we found that the immunoreactive band corresponding to phospho-TFEB (p-TFEB) virtually disappeared in all fibroblast lysates from different donor ages when cultured under starvation conditions (Figure 1D). Instead, a series of lower-migrating bands were observed. Since proteasome-mediated degradation of TFEB has been demonstrated to occur once the proteins enter the nucleus (Keshri et al., 2024; Li et al., 2019), it is conceivable that these smaller bands correspond to proteolytic fragments of dephosphorylated TFEB. To evaluate the transcriptional activity of TFEB, the messenger RNA (mRNA) expression of three different TFEB-target genes, namely, LAMP1, SQSTM1 (p62), and MAP1LC3B (LC3B) were assessed (Figure 1E). The expression of LAMP1 mRNA increased in fibroblast cultures derived from all age donors following the induction of TFEB (starvation conditions). However, no significant differences were found in the transcript level of this gene among the fibroblast cultures of the different donor ages before and after autophagy induction (Figure 1E, left graph). With regard to the SQSTM1 gene, an induction of transcript expression was solely observed for the fibroblast cultures derived from the 38- and 96-year-old donors following induction of TFEB by KRB medium (Figure 1E, middle graph). Therefore, the transcript level of this gene was evidently higher in KRB-cultured fibroblasts from the 96-year-old donor compared with those from the 20-year-old donor. In a similar manner, the expression of MAP1LC3B mRNA increases in response to KRB medium only in the fibroblast cultures derived from donors aged 74 and 96 (Figure 1E, right graph). Consequently, the level of this mRNA was markedly higher in KRB-cultured fibroblasts derived from the 96-year-old donor than in KRB-cultured fibroblasts derived from the 20-year-old subject. Collectively, these data imply that TFEB-mediated transcriptional activation of autophagy did not decline during the process of fibroblast aging. However, further comprehensive transcriptomic analyses are required to determine whether overall TFEB transcriptional activity remains fully intact. Furthermore, the elevated levels of SQSTM1 and MAP1LC3B transcripts in aged fibroblasts (donor of 96 years old) in response to KRB-mediated TFEB induction are likely attributable to the premature nuclear accumulation of TFEB in the rich-medium culturing condition (see Figures 1B,C).

### 3.2 Autophagosome formation is maintained during human fibroblasts aging

The autophagosome protein LC3 was evaluated as a *bona fide* indicator of the formation of autophagosomes (Figure 2A) (Feng et al., 2014; Gómez-Virgilio et al., 2022; Parzych and Klionsky, 2014). The fibroblast cultures were treated for 24 h with 50 µM chloroquine (CQ) to inhibit the fusion between the autophagosome and the lysosome and facilitate the quantification of LC3-positive autophagosomes (Figure 2B). Upon treatment with CQ, the generation of LC3-stained fluorescent foci was evident in fibroblast cultures from all age donors, as demonstrated by confocal microscopy analysis (Figure 2B). However, a substantial decrease in the number of LC3 foci per cell was observed in fibroblasts derived from individuals aged 38 and 74 years, but not in those of the elderly 96-year-old donor (bottom panel). As aged fibroblasts contain overloaded lysosomes (Kim et al., 2018; Tigges et al., 2014), the accumulation of LC3 puncta may be indicative of a state of lysosomal frailty that is uncovered by exposure to CQ. It has been established that LC3 is conjugated to phosphatidylethanolamine in the autophagosome membrane to generate LC3-II during autophagy activation (Feng et al., 2014; Gómez-Virgilio et al., 2022; Parzych and Klionsky, 2014). Therefore, the turnover from LC3-I to LC3-II was analyzed by immunoblotting. Following CQ treatment, the faster-migrating band corresponding to LC3-II clearly accumulated in fibroblasts from all donor ages (Figure 2C). The quantification of the LC3-II/LC3-I ratio indicated that the formation of autophagosomes occurred with similar efficiency among fibroblasts from individuals of varying ages (bottom panel), with the exception of a minor decrease found in fibroblasts from the 74-year-old donor compared with those of the 20-year-old subject. On the other hand, the analysis of autophagic vacuoles by Cyto-ID labeling revealed an increase in their accumulation in response to CQ treatment in fibroblast cultures from donors aged 20 and 38 years, in comparison with their counterparts treated with vehicle only (Figure 2D). Furthermore, it was observed that fibroblasts from older donors (74 and 96 years old) exhibited a greater accumulation of autophagic vacuoles under both vehicle and CQ treatments, in comparison with fibroblasts from younger donors (20 and 38 years old). Taken together, these results imply that the formation of autophagosomes did not decrease but rather occurred with higher efficiency during the aging of fibroblasts.

**FIGURE 2:**
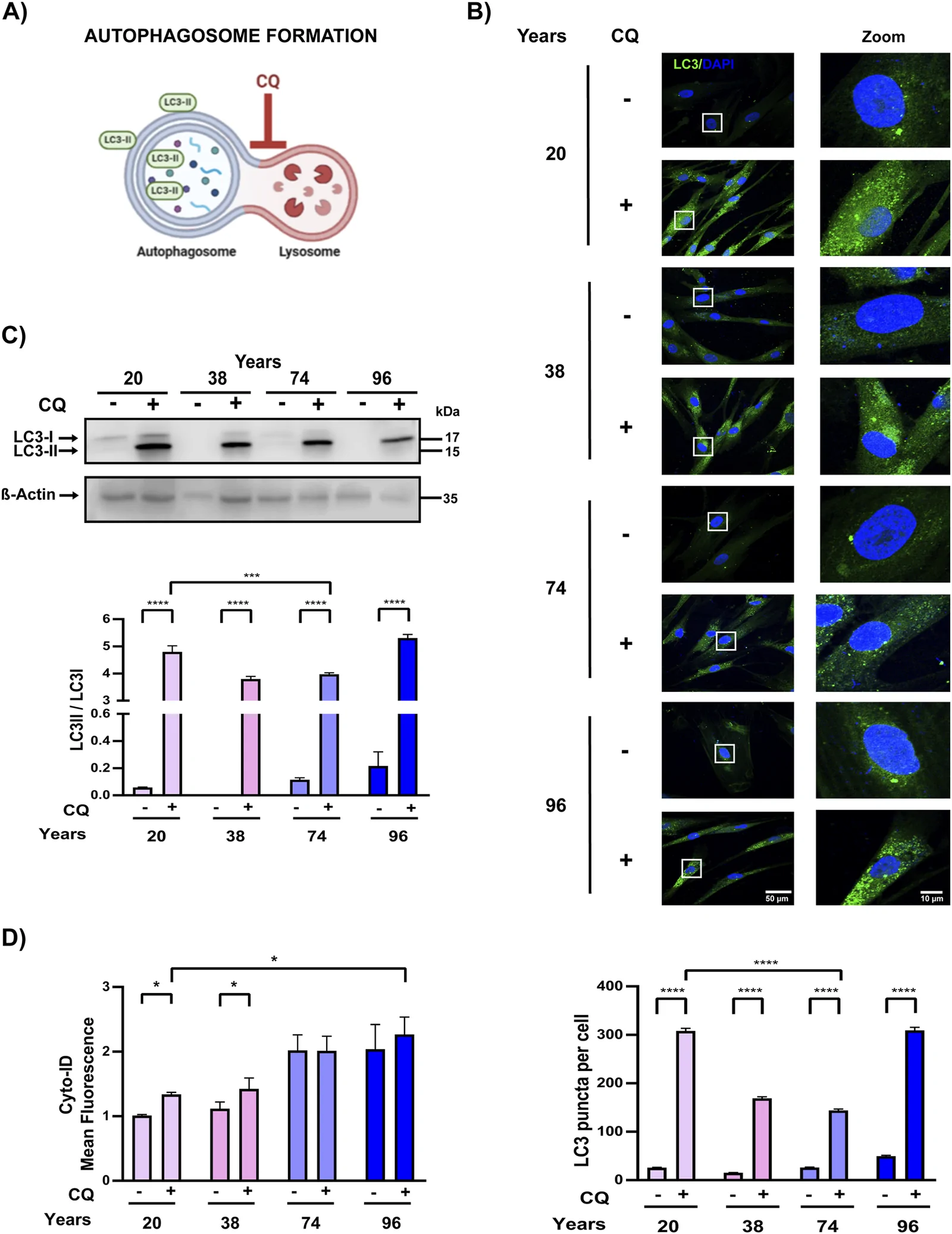
Analysis of autophagosome formation in fibroblast aging. **(A)** Schematical representation of the inhibitory action of CQ on autophagy. **(B)** The indicated fibroblasts from donors of a specified age were seeded on coverslips and treated with 50 CQ for 24 h prior to be immunostained for LC3 and counterstained with DAPI to visualize nuclei. The cells were analyzed by confocal microscopy and typical images from three separate experiments are shown. *Bottom.* Quantification of LC3 puncta in the different fibroblast cultures is shown (see Methods). Data represents the mean ± standard deviation (SD) from three independent experiments (n = 100 cells per condition), with statistical differences determined by Two-Way ANOVA followed Tukey’s *post hoc* test. The results are presented as the mean ± standard error of the mean (SEM), with asterisks denoting significant differences (****p ≤ 0.0001). **(C)** Fibroblast lysates from different donor ages were analyzed by immunoblotting using primary antibodies against LC3 and β-actin (loading control). *Bottom.* Quantification of the LC3-II/LC3-I ratio from 3 independent experiments was carried out using Two-Way ANOVA followed Tukey’s *post hoc* test. The results are presented as the mean ± SEM (***p ≤ 0.001; ****p ≤ 0.0001). **(D)** Fibroblast cultures from donors of different ages were treated with 50 μM CQ or the vehicle alone for 24 h prior to staining with the Cyto-ID autophagy detection kit. The samples were then analyzed by flow cytometry to estimate autophagic vacuoles from three independent experiments (>10,000 cells per experiment) Significant differences were determined by Two-Way ANOVA followed Tukey’s *post hoc* test for comparison between fibroblast cultures from donors aged 20 and 96 years. Significant differences were determined with CQ and Sidak´s *post hoc* test for comparison between fibroblast cultures from donors aged 20 and 38 years. The results are presented as the mean ± SEM, (*p ≤ 0.05) from three independent experiments.

### 3.3 The lysosomal turnover of LC3 and p62 is impaired in aged fibroblasts

The ubiquitin-binding protein p62, also known as sequestosome 1 (SQSTM1), functions as a selective autophagy receptor (Feng et al., 2014; Gómez-Virgilio et al., 2022; Kumsta et al., 2019; Parzych and Klionsky, 2014). As p62 is itself subjected to degradation in autolysosomes, its content increases when autophagy flux is impaired and decreases with proper autophagy activation (Feng et al., 2014; Gómez-Virgilio et al., 2022; Kumsta et al., 2019; Parzych and Klionsky, 2014). Thus, p62 protein was analyzed in fibroblast cultures of varying donor ages treated with CQ to inhibit autophagy flux and monitor its accumulation (Figure 3A). The p62 immunofluorescent staining was barely observed in fibroblasts from all age donors; however, a clear accumulation of p62 fluorescence puncta was observed upon CQ treatment in all fibroblast cultures (Figure 3B and bottom panel). It is worth noting that the number of p62 puncta was significantly lower in CQ-treated fibroblasts from the donor aged 96 years compared with those of the donor aged 20 years. In accordance with the aforementioned results, the p62 protein was found to be virtually absent in lysates of untreated fibroblast from all age donors, with the exception of a faint band observed in 96-year-old fibroblasts (Figure 3C and bottom panel). However, the level of p62 significantly increased in CQ-treated fibroblasts from all age donors (Figure 3C and bottom panel), with a certain decrease found in the fibroblasts of the donor aged 96 years when compared with those of the donor aged 20 years. To strengthen the analysis of autophagy in fibroblast aging, autophagy flux was arrested with Bafilomycin A1 (BafA1), an inhibitor of the V-ATPase pump that blocks autophagosome-lysosome fusion, thereby enabling the quantification of autophagic flux by monitoring the accumulation of LC3-II and p62 in young (20 years old) and old (96 years old) fibroblast cultures. An increase in LC3 and p62 immunofluorescence signals was observed following autophagy inhibition via BafA1 in both fibroblast cultures (Figure 4). Notably, fibroblasts from the older donor exhibited significantly higher basal levels of both markers compared to their younger counterparts, as well as a more pronounced net accumulation following BafA1 treatment (Figures 4B versus Figures 4C). Overall, these data indicate that autophagic flux is not only preserved during human fibroblast aging but operates with high efficacy in 96-year-old fibroblasts, likely functioning as an active compensatory mechanism to cope with age-related cellular stress.

**FIGURE 3:**
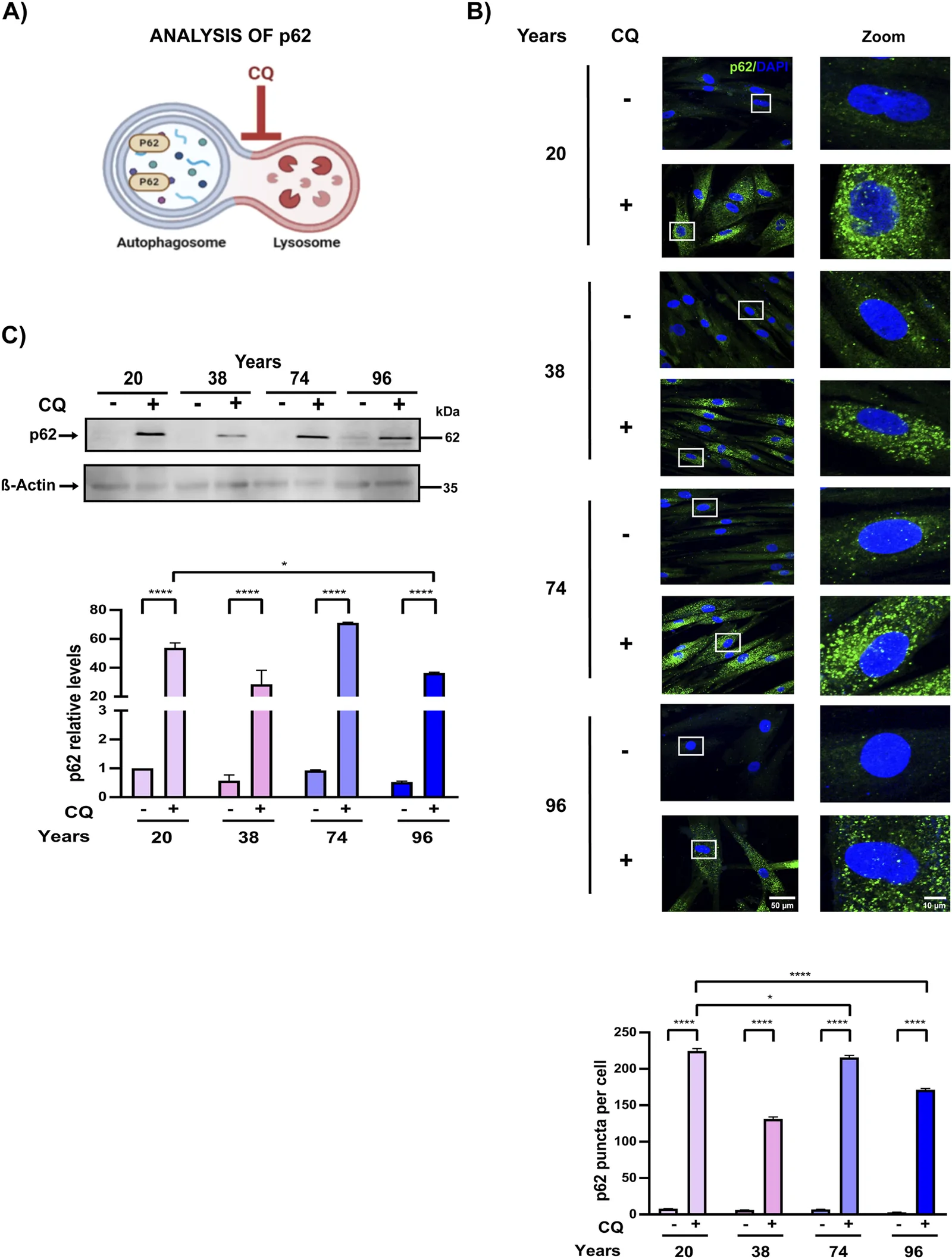
Accumulation of p62 in response to autophagy inhibition in fibroblast cultures from different donor ages. **(A)** The scheme delineates the experimental strategy for analyzing the accumulation of p62 in autophagosomes upon CQ-mediated autophagy inhibition. **(B)** The indicated fibroblast cultures were grown on coverslips and treated with 50 μM CQ or the vehicle alone for 24 h, and then immunostained for p62. Nuclei were labeled with DAPI for visualization prior to confocal microscopy analysis, and typical images from three independent experiments are shown. *Bottom.* The quantity of p62 puncta per cell was determined from three separate experiments (n = 20 cells per experiment), with statistically significant differences being determined by Two-Way ANOVA followed Tukey’s *post hoc* test. The results are presented as the mean ± SEM (*p ≤ 0.05; ****p ≤ 0.0001). **(C)** Lysates from the indicated fibroblast cultures previously treated with 50 μM CQ or the vehicle alone as per B, for 24 h were subjected to Western blotting analysis using antibodies specific to p62 and β-actin (loading control). *Bottom.* The relative protein level of p62 was measured from three independent experiments, with significant differences obtained by Two-Way ANOVA followed Tukey’s *post hoc* test; the results are presented as the mean ± SEM, (**p ≤ 0.01; ****p ≤ 0.0001).

**FIGURE 4:**
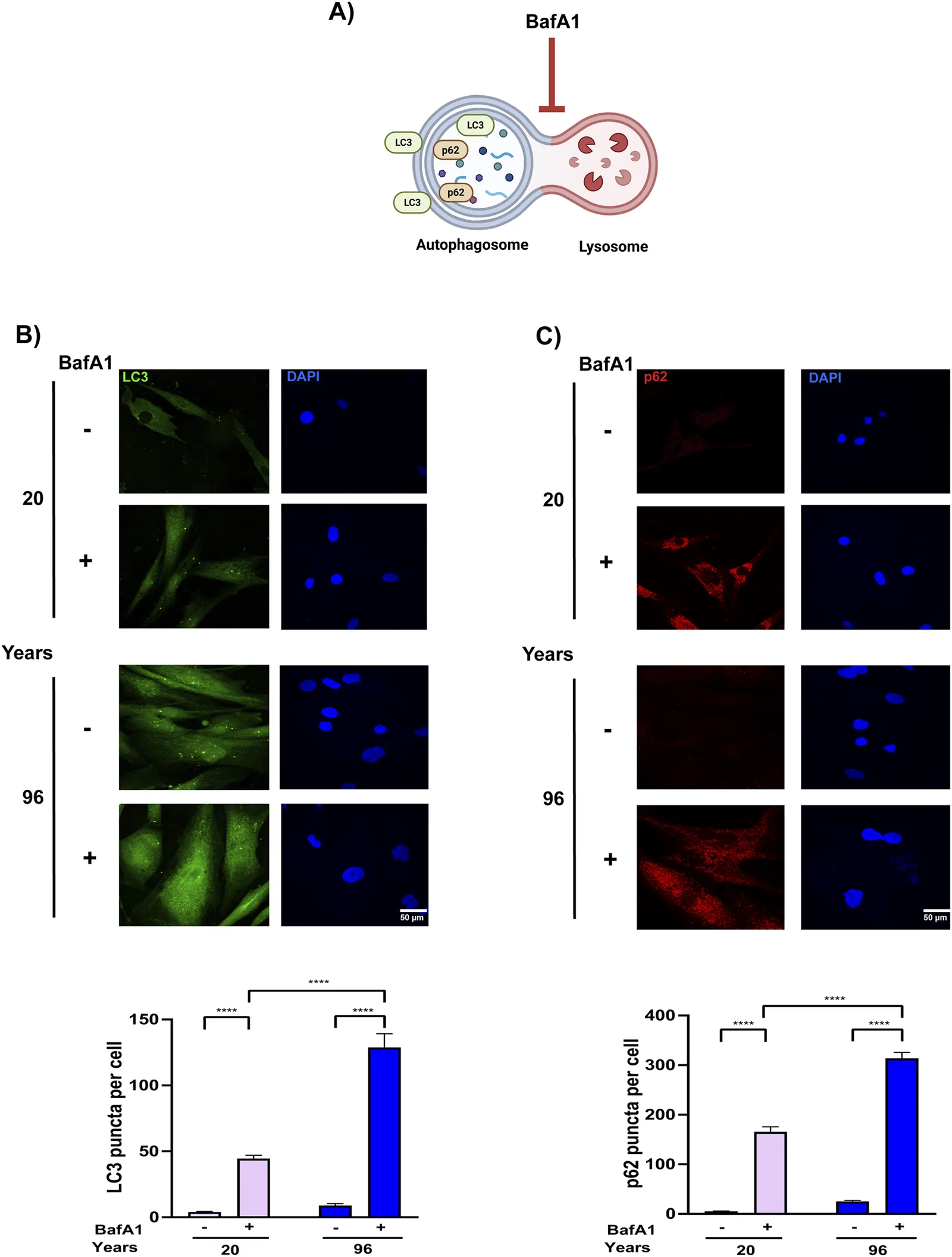
Accumulation of LC3 and p62 in response to BAF1-mediated autophagy inhibition in fibroblast cultures from different donor ages. **(A)** The scheme delineates the experimental strategy for analyzing the accumulation of LC3 and p62 upon BAF1-mediated autophagy inhibition. **(B)** The fibroblast cultures from donors of 20 and 96 years old were grown on coverslips and treated with 50 μM BafA1 or DMSO alone (vehicle) for 12 h, and then immunostained for LC3 and counterstained with DAPI to visualize nuclei. The cells were analyzed by confocal microscopy and typical images from three separate experiments are shown. *Bottom.* Quantification of LC3 puncta in the different fibroblast cultures is shown (see Methods). Data represent the mean ± standard deviation (SD) from three independent experiments (n = 100 cells per condition), with statistical differences determined by Two-Way ANOVA followed Tukey’s *post hoc* test. The results are presented as the mean ± standard error of the mean (SEM), with asterisks denoting significant differences (****p ≤ 0.0001). **(C)** The fibroblast cultures from donors of 20 and 96 yearsold were grown on coverslips and treated with 50 μM BafA1 or DMSO alone (vehicle) for 1|2 h, and then immunostained for p62. Nuclei were labeled with DAPI for visualization prior to confocal microscopy analysis, and typical images from three independent experiments are shown. *Bottom.* The quantity of p62 puncta per cell was determined from three separate experiments (n = 20 cells per experiment), with statistically significant differences being determined by Two-Way ANOVA followed Tukey’s *post hoc* test. The results are presented as the mean ± SEM, (****p ≤ 0.0001).

Given that p62 is involved in the ubiquitin-proteasome system (UPS) and its transcription is subject to regulation (Fu et al., 2021; Xiao et al., 2025), we sought to analyze the turnover of p62 within the lysosome, to avoid interference from other processes/mechanisms. To this end, fibroblasts were cultivated under conditions of starvation and treated with lysosomal function inhibitors (E64D and pepstatin A), to impede autophagy flux at the lysosomal level (Figure 5A). A significant accumulation of LC3-II and p62 was observed in fibroblast lysates from young (20 years old) and middle-aged (38 years old) donors when cultured in KRB medium and treated with lysosomal inhibitors (Figures 5B,C). By contrast, no significant accumulation of the autophagy flux markers LC3-II and p62, was found in fibroblast lysates derived from old-age donors (74 and 96 years old) when were grown under starvation conditions and treated with lysosomal inhibitors (Figures 5D,E). Overall, these findings imply that autophagy flux is indeed impeded at the final stages in aged fibroblasts.

**FIGURE 5:**
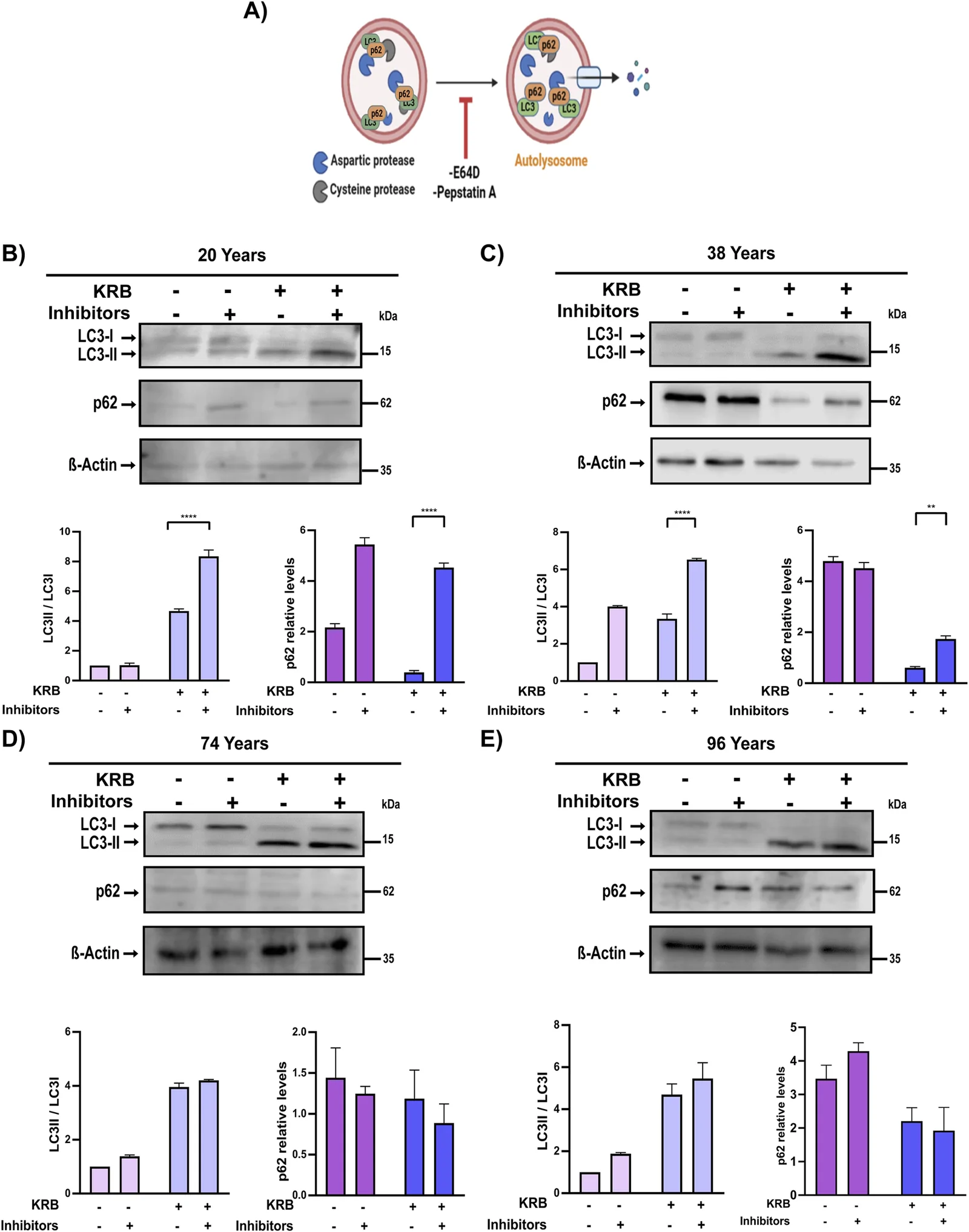
Lysosomal turnover of LC3 and p62 in fibroblast aging. **(A)** The scheme delineates the strategy to analyze the lysosomal turnover of LC3 and p62 upon lysosome activity inhibition by E64d and pepstatin A. **(B–E)** The indicated fibroblast cultures were grown in complete medium or KRB medium (starvation) and treated for 4 h with lysosomal protease inhibitors E64 (10 μg/mL) and pepstatin A (10 μg/mL). Subsequently, the corresponding lysates were subjected to Western blotting analysis using antibodies specific to LC3, p62 and β-actin (loading control). Representative blots from three independent experiments are shown**.** *Bottom panels* **.** The ratio of LC3II/LC3I and the relative level of p62 within lysosomes were estimated from three independent experiments, with significant differences determined by Two-Way ANOVA followed Tukey’s *post hoc* test; the results are presented as the mean ± SEM, (*p ≤ 0.05; ****p ≤ 0.0001).

### 3.4 Confocal microscopy analysis of autophagosome maturation and autophagosome-lysosome fusion in fibroblasts aging

An analysis of the final stages of autophagy was conducted, specifically the maturation of autophagosomes and their subsequent fusion with lysosomes. Autophagosome maturation and sealing is dependent upon the recruitment of the autophagosomal soluble N-ethylmaleimide-sensitive factor activating protein receptor (SNARE) protein STX17 (Itakura et al., 2012; Itakura and Mizushima, 2013). Therefore, the presence of mature autophagosomes was identified by the colocalization between STX17 and LC3 in autophagosomes from young (20 years old) and aged (96 years old) donors (Figure 6A). Fibroblasts were cultured in a condition of starvation and treated with CQ prior to being imaged using confocal microscopy. The number of LC3 puncta colocalizing with STX17 was found to increase significantly in young fibroblast samples upon starvation, compared with those cultured in rich medium, which indicates enhance spatial proximity between the two fluorescence signals (Figure 6C). This increase was confirmed by Pearson correlation values, which ranged from 0.35 to 0.63 for culturing in rich medium and KRB medium, respectively. In contrast, colocalization between STX17 and LC3 did not increase when old fibroblasts were cultured under starvation conditions, compared with those cultured in rich medium. As indicated by Pearson correlation values of 0.42 versus 0.39 for rich medium and KRB medium, respectively (Figure 6C). Further object-based analysis using ImageJ demonstrated consistency with the Pearson coefficient (data not shown). One of the final steps in autophagy is the fusion between the autophagosome and the lysosome. Thus, the presence of membranous autophagosomal structures showing colocalization of LC3 with the lysosomal membrane protein LAMP1 was utilized as an indicator of the frequency of the fusion between the autophagosome and the lysosome (Figure 6B). Fibroblasts from young (20 years old) and aged (96 years old) donors were cultured under conditions of starvation and treated with lysosome inhibitors (E64D and pepstatin A), to impede autophagy flux at the lysosomal level and accumulate fused autophagosome-lysosome structures prior to confocal microscopy analysis. A substantial rise in the number of LC3 puncta colocalizing with LAMP1 was observed in lysosome inhibitors-treated young fibroblasts upon culturing in starvation conditions compared to untreated young fibroblasts (Figure 6D; Pearson correlation values of 0.49 and 0.67 for culturing in rich medium and KRB medium, respectively). Conversely, a marginal increase in the number of LC3 puncta colocalizing with LAMP1 was observed in aged fibroblasts upon KRB-mediated autophagy induction (see Figure 6D). These data indicate spatial proximity and probably association between mature autophagosome and lysosome. This observation was corroborated by Pearson’s correlation coefficients, which yielded values of 0.54 and 0.60 for culturing in rich medium and KRB medium, respectively. Further object-based analysis using ImageJ demonstrated consistency with the Pearson coefficient (data not shown). To expand the insights on these two autophagy critical proteins in the aging process of fibroblasts, their protein levels were evaluated by immunoblotting. No significant changes were observed for LAMP1 among the fibroblast cultures from donors of varying ages, while STX17 was found to be significantly augmented in the fibroblasts derived from the 95-year-old donor (Figure 6E and right panels). Collectively, these results suggest a decline in autophagy flux at its late stages during the aging process of fibroblasts.

**FIGURE 6:**
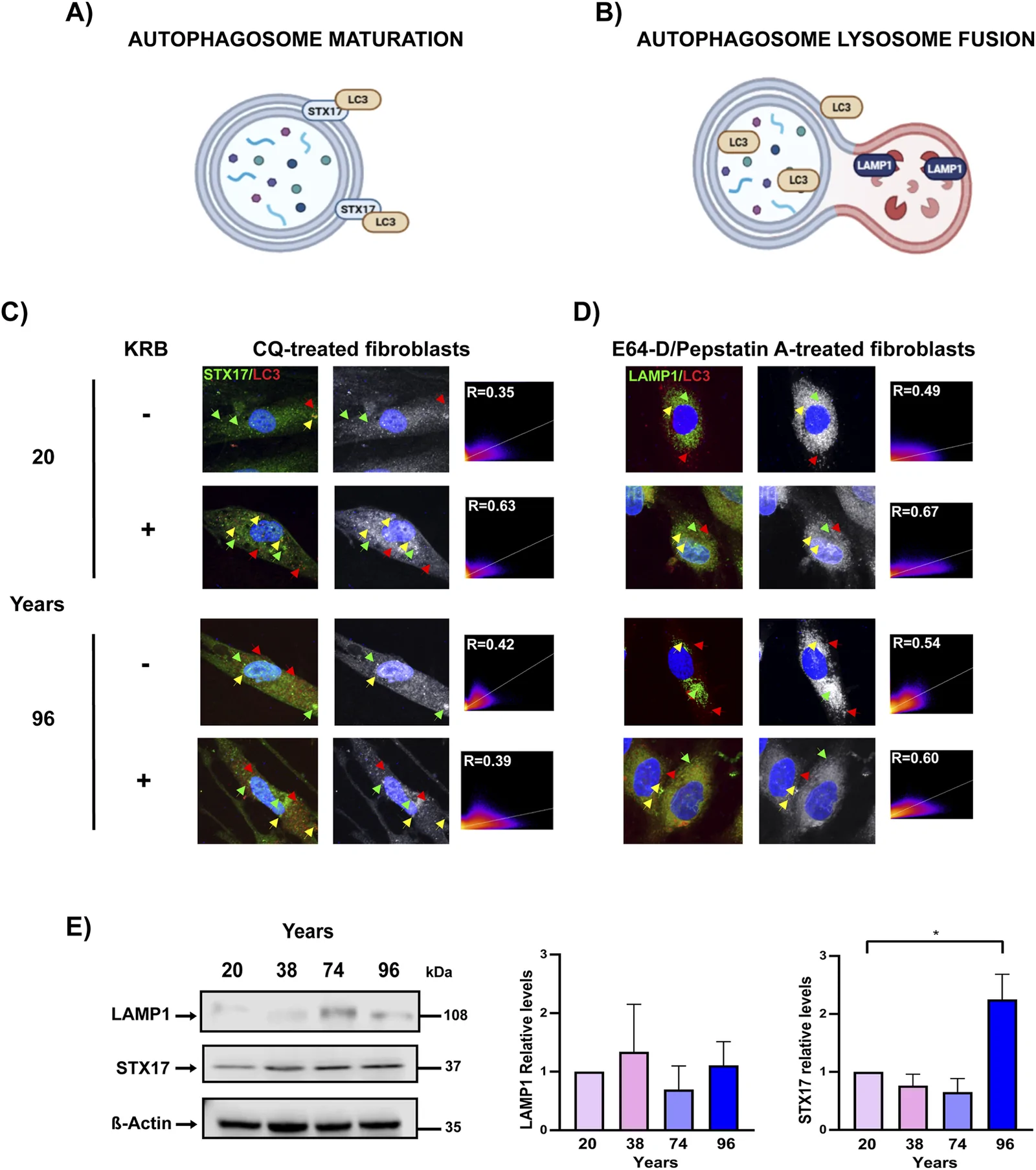
Impairment of autophagosome maturation and autophagosome-lysosome fusion in during aging of fibroblast. **(A,B)** The schemes delineate the strategy to evaluate the maturation of autophagosomes by the colocalization of LC3 and STX17 in autophagosomes **(A)**, and the fusion between autophagosomes and lysosomes by the colocalization of LC3 and Lamp1 **(B)**. **(C)** Fibroblast cultures from two donors aged 20 and 96 years were grown on coverslips and treated with 50 mM CQ or the vehicle for 24 h, prior to being incubated in complete medium or KRB medium (starvation) for 60 min. The cells were then subjected to immunostaining using antibodies specific to LC3 and STX17 and counterstained with DAPI for the visualization of nuclei. Typical confocal images from three independent experiments are shown. To quantify STX17-LC3 colocalization, the Pearson correlation coefficient (R) was calculated from a pooled sample of 45 cells per experimental condition using NIS Elements software (Nikon). Green arrows, STX17 punctuate; red arrows, LC3 punctate; yellow arrows, STX17/LC3 colocalizing punctuate. **(D)** Fibroblast cultures from donors aged 20 and 96 years were seeded on coverslips, grown in complete medium or KRB medium (starvation) for 4 h in the presence of lysosomal protease inhibitors E64 (10 μg/mL) and pepstatin A (10 μg/mL). The fibroblasts were then subjected to double immunostaining for LC3 and LAMP1 using the corresponding primary antibodies. Counterstaining with DAPI for nuclei visualization was carried out prior to confocal microscopy analysis. Typical confocal images from three independent experiments are shown. Colocalization between LAMP1 and LC3 was assessed by determining the Pearson correlation coefficient. R values represent pooled data from 45 individual cells per experimental condition, analyzed via Nikon’s NIS Elements software. Green arrows, LAMP1 punctuate; red arrows, LC3 punctate; yellow arrows, LAMP1/LC3 colocalizing punctuate. **(E)** Lysates from fibroblast cultures derived from donors of different ages were analyzed by Western blotting using primary antibodies against STX17, LAMP1 and β-actin (loading control). Typical immunoblots from three independent experiments are shown. Right. The relative levels of LAMP1 and STX17 were estimated, with significant differences determined by Two-Way ANOVA followed Tukey’s *post hoc* test. The data are presented as the mean ± SEM, (*p ≤ 0.05) from three independent experiments.

### 3.5 Altered lysosomal morphology and function in aged fibroblast

As the autophagosome-lysosome axis appears to be impaired in aged fibroblasts, an evaluation of the lysosomal morphology and function was conducted in fibroblasts from different age donors grown on normal (rich medium) or starvation (KRB medium) conditions. A significant increase in the lysosomal abundance was evidenced by LysoBrite staining in fibroblasts from the oldest donor (96 years old) under both culturing conditions, in contrast to those of donors aged 20 and 38 years (Figures 7A,B). Remarkable, enlarged lysosomes were found in all fibroblast cultures following their growth in starvation conditions, with the most expanded lysosomes being observed specifically in fibroblasts derived from older donors (74 and 96 years of age) (Figures 7A,C). Furthermore, lysosomal activity was found to be augmented in fibroblasts from donors of all ages when autophagy was induced by culturing in KRB medium, with the exception of fibroblasts from the oldest donor (96 years), which exhibited the greatest basal activity of lysosomes but were unresponsive to autophagy induction (Figure 7D). Consistent with this finding, the accumulation of lipofuscin (oxidized lipid and protein complexes) was higher in fibroblasts from donor aged 96 years (Figure 7E). Furthermore, the induction of autophagy that led to a decrease in lipofuscin levels in fibroblast cultures from donors aged 20 and 38 years, was insufficient to fully counteract the accumulation of lipofuscin in fibroblasts samples from older donors (74 and 96 years of age) (Figure 7E). These findings suggest that enlarged lysosomes from aged fibroblasts are overloaded with waste material and damaged cellular components, and that the lysosomal capacity to remove this waste is impaired even after autophagy induction.

**FIGURE 7:**
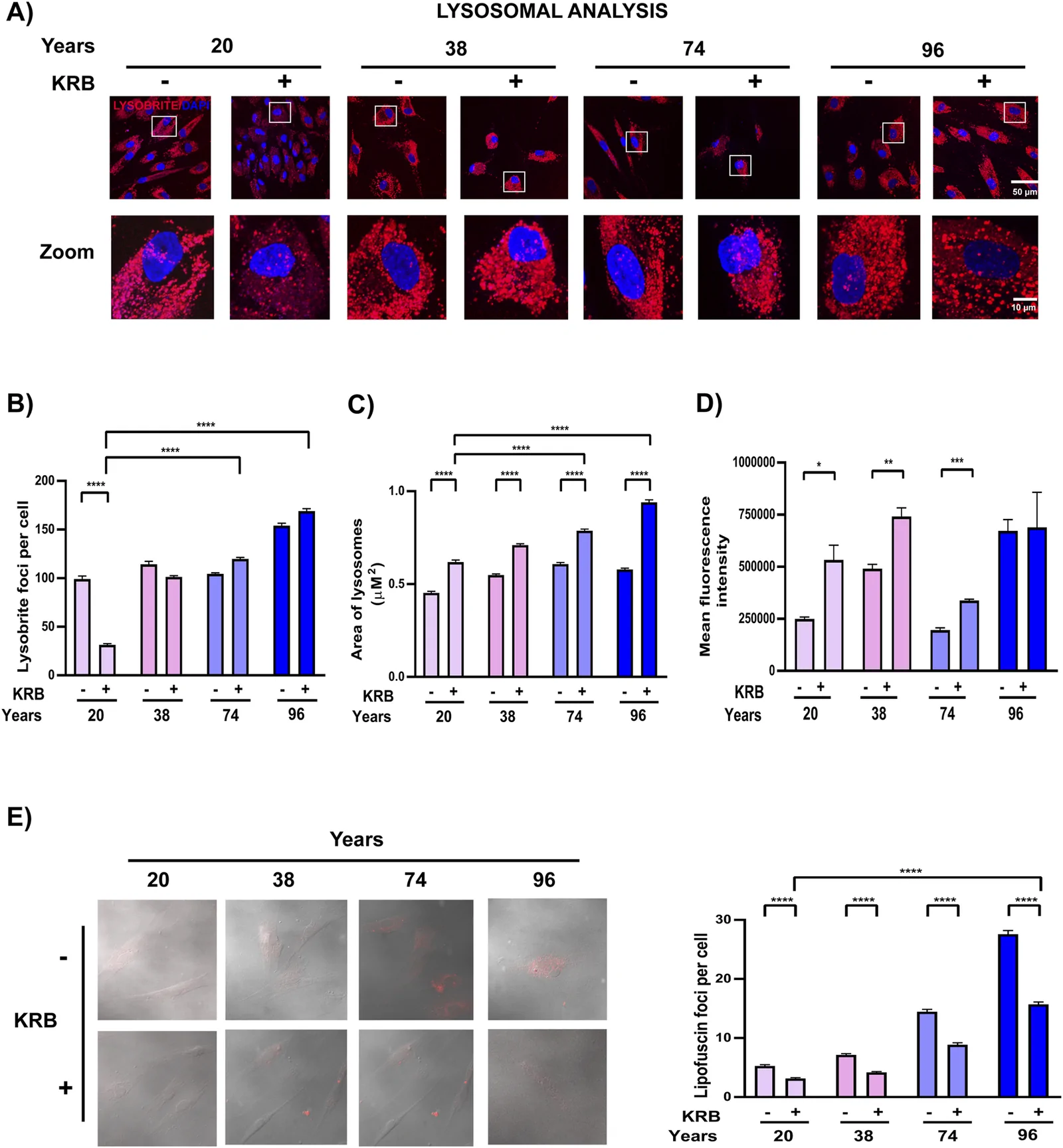
Analysis of lysosome morphology and function in the aging of fibroblasts. **(A)** Fibroblast cultures from donors of a range of ages were grown on coverslips and further staining with LysoBrite and Hoechst, to decorate lysosomes and nuclei, respectively, prior to being subjected to confocal microscopy analysis. Typical images from three independent experiments are shown. **(B,C)** The number of lysosomes per cell and the lysosomal area were quantified in the fibroblast cultures of the indicated donors’ ages (n = 100 cells per experimental condition and from each of the three independent experiments), and significant differences were determined by Two-Way ANOVA followed Tukey’s *post hoc* test. The data are presented as the mean ± SEM, (****p ≤ 0.0001) from three independent experiments. **(D)** The lysosomal intracellular activity was assessed in fibroblast cultures derived from donors of varying ages by staining the cells with a Lysosomal Intracellular Activity Kit, and further analysis by flow cytometry. Control cells (20 years donor) treated with 200 nM Bafilomycin A1 for 24 h were utilized as a negative control. The quantification from three independent experiments was carried out (10,000 events per experimental condition and from each independent experiment), and statistical significance was determined by Two-Way ANOVA followed Tukey’s *post hoc* test, the results are presented as the mean ± SEM from three independent experiments (*p ≤ 0.05; **p ≤ 0.01; ***p ≤ 0.001). **(E)** The analysis of lipofuscin aggregates was conducted in fibroblast cultures derived from donors of various ages. These cells were grown on cover slips in either MEM or KRB medium (starvation) prior to being subjected to confocal microscopy analysis at 550–650 nm. Representative images from three independent experiments are shown. *Right*. The number of lipofuscin foci per cell was estimated from three independent experiments, with significant differences determined by Two-Way ANOVA followed Tukey’s *post hoc* test, the results are presented as the mean ± SEM, (****p ≤ 0.0001).

## 4 Discussion

In this study, we conducted an analysis of autophagy in aging using a cell system consisting of primary cultures of dermal fibroblasts derived from healthy individuals of different ages (20–96 years). This aging cell model is well-established and widely employed for studying the cellular and molecular changes associated with aging (Brun et al., 2016; Cristofalo et al., 1998; Kim et al., 2018; Rorteau et al., 2022; Tigges et al., 2014; Tsitsipatis et al., 2022; Zorin et al., 2019.) Autophagy exhibits a close association with the aging process, yet despite the extensive research conducted on this process, there is a lack of studies, specifically on autophagic flux, which is required to understand its various stages. Accordingly, we proceeded to analyze the dynamics of autophagy, including its transcriptional control through the master transcription factor of autophagy and lysosomal biogenesis, TFEB; the formation and maturation of autophagosomes and their fusion with lysosomes by confocal microscopy analysis; as well as lysosomal function. Our findings indicate that TFEB activation and the formation of autophagosomes remained unaltered throughout the aging process. However, the final steps of autophagy, including autophagosome maturation and fusion with lysosomes, as well as lysosomal function, appears to be dysregulated in aged fibroblasts.

Unexpectedly, the transcriptional regulation of autophagy by TFEB, which induces the expression of autophagy and lysogenesis genes (Abokyi et al., 2023; Di Malta et al., 2019), appears to be enhanced in aged fibroblasts. This finding was evidenced by the higher nuclear accumulation of TFEB and the upregulation of TFEB target genes (SQSTM1 and MAP1LC3B) in fibroblasts from a subject aged 96 years under starvation conditions compared to fibroblasts from a subject aged 20 years. However, these findings indicate the preservation of selective downstream responses rather than fully intact overall TFEB activity. During starvation, TFEB undergoes extensive dephosphorylation, enabling its nuclear translocation and subsequent proteasomal degradation (Keshri et al., 2024; Li et al., 2019). Thus, our analysis of TFEB by immunoblotting, which revealed a series of shorter peptides in fibroblast subjects at different ages cultures in rich medium that were no longer present in KRB medium-mediated starvation, is consistent with the notion that proteasomal degradation of TFEB occurs in a similar fashion during fibroblast aging. Contrary to this, the unexpected nuclear accumulation of TFEB found in aged fibroblasts (a 96-year-old donor) cultured in a non-starvation condition may be an anticipated signaling mechanism to counteract the impaired autophagy flux and/or lysosomal dysfunction found in these cells (see below). In support of this notion, it has been demonstrated the activation of TFEB due to lysosomal homeostasis imbalance during aging (Curnock et al., 2023; Napolitano and Ballabio, 2016). TFEB induces the expression of genes involved in lysosomal biogenesis and autophagy, thereby enhancing lysosomal clearance and restoring cellular homeostasis (Martina et al., 2012). Furthermore, TFEB plays a protective role in maintaining homeostasis and mounting the cellular response to stress (Guerrero-Navarro et al., 2025; Wang et al., 2020). The collective evidence indicates that nuclear trafficking and transcriptional activity of TFEB persist in aged fibroblasts. While increased nuclear TFEB in 96-year-old fibroblasts likely reflects a compensatory response to impaired autophagy flux, alternative mechanisms may contribute. For instance, altered mTORC1- or ERK-mediated phosphorylation of TFEB, could prevent its normal cytoplasmic retention under nutrient-rich conditions (Martina et al., 2012; Napolitano and Ballabio, 2016). Additionally, age-dependent decline in CRM1-mediated nuclear export could trap TFEB in nucleus. Further experiments are required to address these hypotheses.

Interestingly, autophagy flux, as monitored by the accumulation of LC3-II and p62 in CQ-treated cells, exhibited comparable or discrete differences among fibroblast from subjects of different ages. In alignment with this finding, a previous study on fibroblast aging reported that the basal autophagic flux in aged dermal fibroblasts is closely comparable to that of young fibroblasts, as measured by LC3 and p62 protein level and LC3-I to LC3-II turnover (Kim et al., 2018). By contrast, proteomic analysis revealed increased autophagy in aged fibroblasts, as evidenced by increased levels of various autophagy proteins, including ATG5, ATG7, ATG16L1, and Beclin-1, but not p62 (Tsitsipatis et al., 2022). Furthermore, the conversion of LC3-I to LC3-II was found to be higher in aged fibroblasts in this study (Tsitsipatis et al., 2022). Nonetheless, it is important to note that LC3 and p62 have functions beyond autophagy. P62 is a multifunctional protein involved in nutrient sensing, oxidative stress, and proteasomal degradation (Fu et al., 2021; Liu et al., 2016; Xiao et al., 2025), while LC3 participates in the integrated stress response (Martina et al., 2016), phagocytosis (Heckmann and Green, 2019) and endocytosis (Chidambaram and Chinnathambi, 2025; Heckmann et al., 2019). Therefore, we proceeded to analyze the lysosomal-dependent turnover of these proteins in fibroblast cultures treated with lysosomal inhibitors to specifically quantify their pool engaged in autophagy. This approach revealed a clear decay in the accumulation of both LC3-II and p62 in aged fibroblasts (donors of 74 and 96 years old), compared with fibroblast cultures from young subjects (20 and 38 years old). This implies that the late steps of autophagy are indeed impaired in aged cells. In line with this idea, we found a decrease in the number of mature autophagosomes, identified by the colocalization of LC3 and STX17 in fibroblasts cultured under starvation and treated with CQ. The recruitment of STX17 to sealed autophagosomes is critical for their maturation (Itakura et al., 2012; Itakura and Mizushima, 2013). Furthermore, the number of autophagosome-lysosome fusion events diminished in aged fibroblasts (subject of 96 years old) compared to young fibroblasts (donor of 20 years old), as determined by the colocalization of LC3 and the lysosomal membrane protein LAMP1. Confocal microscope data are indicative of spatial proximity and probably association between mature autophagosome and lysosome.

As lysosomes play a crucial role in the completion of autophagic flux (Aman et al., 2021; Tan and Finkel, 2023), their content, morphology, and function were assessed in the aging cell system. A direct correlation was found between the advanced age of donors and increased number and size of lysosomes, with statistically significance between fibroblasts from donors aged 20 and 96 years. In starvation, the area of lysosomes was greater than that observed in normal culture conditions in all fibroblast cultures; however, the presence of enlarged lysosomes was a distinctive feature of aged fibroblasts (donors of 96 years old). In line with this, lysosomal enlargement has been found to be associated with aging in *C. elegans* (Sun et al., 2020). Specifically, enlarged lysosomes exhibited reduced acidity, decreased motility, and impaired degradative capacity (Sun et al., 2020). It is thought that that accumulation of damaged organelles and protein aggregates during aging increases the demand for autophagy, thereby promoting lysosomal enlargement (Liu et al., 2026), as evidence in *Drosophila* muscle remodeling (Murakawa et al., 2020). This enlargement is often accompanied by the buildup of waste products and lipofuscin, a non-degradable autofluorescence pigment formed as a byproduct of incomplete lysosomal digestion (Escrevente et al., 2021). In align with the notion of expanded dysfunctional lysosomes present in aged cells, we observed accumulation of lipofuscin in fibroblasts from aged subjects (74 and 96 years old) even after the induction of lysogenesis by starvation, compared with fibroblasts from younger subjects (20 and 38 years old).

### 4.1 Limitation of the study

The use of a series of dermal fibroblast primary cultures from subjects of varying ages facilitates the recreation of autophagy flux during the aging process, thereby ensuring a high degree of internal validity. However, it is acknowledged that the employment of a single donor per age group limits the ability to fully account for inter-individual biological variation. This limitation is of relevance when evaluating autophagy flux, a multi-step and tightly regulated pathway, which may be influenced by the metabolic or genetic baseline of each individual. Consequently, future studies incorporating biological replicates from additional donors within the same age cohorts will be necessary to confirm whether the impairment of autophagy at its late stages constitutes a hallmark of fibroblast aging.

## 5 Conclusion

In summary, the use of a cellular system based on primary fibroblast cultures from subjects of different ages (20–96 years) enabled the analysis of autophagy flux during aging. The initiation of autophagy appears to occur normally during fibroblast aging, including the activation of the autophagy and lysosomal biogenesis master transcription factor TFEB, and the formation of autophagosomes. However, the final steps of autophagy were found to be apparently altered during fibroblast aging, including the maturation of autophagosomes and the fusion between autophagosomes and lysosomes. Furthermore, we provided evidence that fibroblasts from aged subjects contain enlarged, dysfunctional lysosomes, which correlates with and could partially account for the observed decline in autophagic flux. This study provides a rationale for promoting healthy aging by targeting autophagy flux at its final steps and enhancing lysosomal function. Promising approaches include TFEB activators to stimulate lysosomal biogenesis via the CLEAR network (Palmieri et al., 2011), mTOR inhibitors (e.g., rapamycin) to restore autophagic flux, and lysosomal acidification enhancers (e.g., small molecules or acidic nanoparticles) to enhance enzymatic activity of lysosomes (Steinhauser et al., 2026). Evaluating these specific interventions in aged models will be essential to validate their therapeutic potential against impaired autophagy.

## Data Availability

The data used to support the findings of this study are available from the corresponding author upon reasonable request.
